# Stress Prediction for Distributed Structural Health Monitoring Using Existing Measurements and Pattern Recognition

**DOI:** 10.3390/s18020419

**Published:** 2018-02-01

**Authors:** Wei Lu, Jun Teng, Qiushi Zhou, Qiexin Peng

**Affiliations:** Harbin Institute of Technology (Shenzhen), Shenzhen 518055, China; qiushi12281989@126.com (Q.Z.); qiexinpeng@stu.hit.edu.cn (Q.P.)

**Keywords:** structural health monitoring, stress capturing, pattern recognition, stress prediction, Shenzhen Bay Stadium

## Abstract

The stress in structural steel members is the most useful and directly measurable physical quantity to evaluate the structural safety in structural health monitoring, which is also an important index to evaluate the stress distribution and force condition of structures during structural construction and service phases. Thus, it is common to set stress as a measure in steel structural monitoring. Considering the economy and the importance of the structural members, there are only a limited number of sensors that can be placed, which means that it is impossible to obtain the stresses of all members directly using sensors. This study aims to develop a stress response prediction method for locations where there are insufficent sensors, using measurements from a limited number of sensors and pattern recognition. The detailed improved aspects are: (1) a distributed computing process is proposed, where the same pattern is recognized by several subsets of measurements; and (2) the pattern recognition using the subset of measurements is carried out by considering the optimal number of sensors and number of fusion patterns. The validity and feasibility of the proposed method are verified using two examples: the finite-element simulation of a single-layer shell-like steel structure, and the structural health monitoring of the space steel roof of Shenzhen Bay Stadium; for the latter, the anti-noise performance of this method is verified by the stress measurements from a real-world project.

## 1. Introduction

Structural stress distribution is a critical and directly-measurable parameter to evaluate structural safety [1] in which monitoring and prediction of the stress distribution of real-world structures is key to the application of structural health monitoring. A stress development prediction method was proposed to predict the stress development trend and provide a deterioration assessment for existing bridges [1]. Long-term monitoring data of dynamic strain were used for fatigue life assessment of steel bridges, where the strain measurement data from the Tsing Ma Bridge were used to verify the method [2]. The maximum stress of beam structures was measured during monitoring, and it was compared with the allowable stress and used to assess structural safety [3]. For such stress prediction methods, the critical problem is that the number of the sensors for stress monitoring is limited, although there is some research on the optimal placement of sensors to control the number of sensors in a reasonable range and ensure that the sensors provide the greatest efficiency. This is because the installation of sensor systems is expensive. Zhang proposed an optimal multi-type sensor placement method to reconstruct the structural responses. The method considered the measurement of structural response and was limited to a few locations [4]. In other words, sensors are arranged only on some key components, and then the stress of the entire structure is identified by the analysis method.

Based on the limited number of stress measurements, the prediction methods for stress distribution were researched and developed. A sensor-free stress estimation model was proposed to estimate the stress distribution of steel beam structures. The model considered the uncertain loads to which the building structure was exposed [5]. A Gaussian particle filtering algorithm was used to predict the dynamic extreme stress based on the monitoring data [6]. A Kalman filter method with a dynamic model of the structure was used to estimate the power spectral density of stresses at unmeasured locations based on the measurements at the limited locations [7]. A model-based state estimator was proposed to predict the stress measurements at arbitrary locations in the structure based on acceleration measurements [8,9,10]. Pattern identification was used to estimate the structural stresses based on stress measurements and displacement measurements [11]. Xu validated the effectiveness and practicability of the proposed multi-type sensor placement and response reconstruction method according to the good agreement of the reconstructed multi-type responses between the numerical studies and the experimental investigations [12]. Lu proposed an improved pattern recognition method, which selected more than one best pattern and used weighting parameters to obtain the synthesized estimated stress values [11]. Zhang presented a restoring method for spatial structural stress monitoring. That method can avoid the missing data using correlation among the measuring points [13]. It is obvious that stress monitoring is important for the structural safety assessment, and there is much research regarding the structural safety assessment using stress measurements and the prediction method for the stress values at the sensor-free placements. However, such methods just considered how to realize the stress prediction, where the prediction processes were all concentrated; in other words, such methods may be unusable if the measurements were partially missed or the central data-processing centre breaks down.

Some research has been based on function dispersion. Smarsly proposed an analytical redundancy approach to decentralized sensor fault detection by using artificial neural networks [14]. Sim presented a decentralized data aggregation approach for system identification based on the random decrement technique, which was used for wireless sensor networks and demonstrated its effectiveness by the accuracy of estimated modal properties and wireless data communication [15]. Lynch developed intelligent data-processing nodes by setting a signal-processing algorithm into sensors. This approach has been applied in the Alamosa Canyon Bridge in New Mexico [16]. Leng divided petroleum pipe software into two subsystems: a data management system and a data analysis system. The two systems work concurrently to complete a second pre-processing, data storage, and data analysis [17]. Gao [18] established a powerful structural health monitoring hardware platform and identified stress by a kind of distributed data acquisition system called ‘small concentrated’. Inaudi [19] introduced the monitoring of data standard management considering the difficulty in storing mass data. The research developed the decentralized functions in condensing mass measurements, data acquisition, and data pre-processing, where the effectiveness has already been proved partially. Furthermore, the structural health monitoring methods should be developed based on such decentralized functions to reduce the potential risks caused by sensor failure and to improve the operation efficiency caused by function concentration.

A stress prediction method in distributed processes is proposed. It is based on the limited stress measurements and pattern recognition method. The aim of the proposed method is to develop a stress prediction method that can be adaptive to the distributed sensor networks accompanying the decentralized functions. Here, we first propose a distributed algorithm based on pattern recognition, where the number of sensors and patterns is considered. Furthermore, simulation of the shell-like structure is performed to verify the effectiveness of the proposed method. In addition, measurements of a real-world structure are used to prove the proposed method by considering noise influences.

## 2. Distributed Algorithm Based on Pattern Recognition

Compared with only one pattern library using all the measurements, there are multiple pattern libraries that recognize stress in monitoring positions to achieve distributed operation. If one pattern library is simply being divided into some subparts, it can be helpful for improving the calculation efficiency, but not for precise recognition.

In this paper, the process of a distributed computing method based on pattern recognition is to establish multiple pattern libraries to recognize the same single pattern, and there are still two improved aspects: (1) information optimised from different pattern libraries, which is the optimization of the number of sensors, and (2) an optimal amount of fusion patterns chosen during a distributed-computing process and final fusion computation. The steps to the method are as follows: (1) sensors are divided into m subgroups; therefore, there will be subpattern-recognizing libraries and subtesting libraries; (2) for each subpattern recognition library and subtesting library, the optimal number of sensors is chosen for the calculation and fusion algorithm patterns; and (3) optimised fusion algorithm patterns are gathered into one total pattern library. Then, the stress prediction of unknown positions based on distributed computing and the pattern recognition method can be completed.

### 2.1. Measurements and Response to Be Recognized

The measurements collected from sensors can be represented as X:

(1) $$X=\left[X_{1}\;\;X_{2}\;\cdots \;X_{i}\;\cdots \;X_{n}\right]$$

where $X_{i}$ is the time series of the measurement collected from the ith sensor.

The response to be recognized can be represented as Y.

### 2.2. Distributed Subsets of Measurements

The measurements X can be divided into m subsets of measurements, which can be represented as $X_{1}$, $X_{2}$,…, $X_{i}$,…, $X_{m}$. The subset of measurements $X_{i}$ is represented as:

(2) $$X_{i}=\left[x_{i1}\;,\;x_{i2}\;,\cdots \;x_{ij}\;\cdots ,\;x_{ip_{i}}\right]$$

(3) $$\sum_{i=1}^{m}p_{i}=n$$

where i=1 , 2 , 3 ,⋯ ,m; $P_{i}$ is the number of measurements in the ith subset of measurements. $x_{ij}$ is the jth measurements in the ith subset of measurements.

### 2.3. Pattern Recognition Based on a Subset of Measurements

#### 2.3.1. Sequenced Responses Based on the Correlation

The finite-element model of the structure is built first. It is used to calculate the structural responses at the locations of sensors and the location to be recognized. The calculated structural responses at the location of sensors and location to be recognized are represented as $x_{ij}$ and y, respectively, which has Q time steps and can be used to give the correlations between the responses at the location of sensors and the response at the location to be recognized. The correlation between the response at the location of sensors and the response at the location to be recognized is represented as $\gamma_{x_{ij}y}$:

(4) $$\gamma_{x_{ij}y}=\frac{\sum_{q=1}^{Q}\left(x_{ij}(q)-{\bar{x}}_{ij})(y(q)-\bar{y}\right)}{\sqrt{\sum_{q=1}^{Q}{\left(x_{ij}(q)-{\bar{x}}_{ij}\right)}^{2}}\sqrt{\sum_{q=1}^{Q}{\left(y(q)-\bar{y}\right)}^{2}}}$$

The responses included in the ith subsets $X_{i}$ can be sequenced by the value of correlation $\gamma_{x_{ij}y}$. The sequenced measurements $X_{i}$ can be represented as $X_{i}^{\prime }$:

(5) $$X_{i}^{\prime }=\left[x_{i1}^{\prime }\;,\;x_{i2}^{\prime }\;,\;\cdots \;x_{ij}^{\prime }\;\cdots \;,\;x_{ip_{i}}^{\prime }\right]$$

where $\gamma_{x_{i1}^{\prime }y}>\gamma_{x_{i2}^{\prime }y}>\;\cdots \;>\;\gamma_{x_{ij}^{\prime }y}\;>\;\cdots \;>\gamma_{x_{ip_{i}}^{\prime }y}$.

#### 2.3.2. Error Analysis on Different Numbers of Sensors and Patterns

The input of the traditional pattern recognition method is $X_{i}^{\prime }$, and the output of the traditional pattern recognition method is ${\hat{Y}}_{i}$.

If the number of sensors is considered, the input of the advanced pattern recognition method can be $X_{i1}^{\prime }$, $X_{i2}^{\prime }$,…, $X_{ij}^{\prime }$,…, $X_{ip_{i}}^{\prime }$, respectively:

(6) $$X_{i1}^{\prime }=\left[X_{i1}^{\prime }\right]$$

(7) $$X_{i2}^{\prime }=\left[X_{i1}^{\prime }\;,\;X_{i2}^{\prime }\right]$$

(8) $$X_{ij}^{\prime }=\left[X_{i1}^{\prime }\;,\;X_{i2}^{\prime }\;,\cdots ,\;X_{ij}^{\prime }\right]$$

(9) $$X_{ip_{i}}^{\prime }=\left[X_{i1}^{\prime }\;,\;X_{i2}^{\prime }\;,\cdots \;X_{ij}^{\prime }\;\cdots \;X_{ip_{i}}^{\prime }\right]$$

where $j=1\;,\;2\;,\;3\;,\;\cdots \;,\;p_{i}$; j represents the number of sensors in ith subset.

If the number of recognized patterns is considered, the output of the advanced pattern recognition method can be ${\hat{Y}}_{i1}$, ${\hat{Y}}_{i2}$,…, ${\hat{Y}}_{ij}$,…, ${\hat{Y}}_{ip_{i}}$, respectively:

(10) $${\hat{Y}}_{ij}=\left[{\hat{Y}}_{ij1}\;,\;{\hat{Y}}_{ij2}\;,\;\cdots \;{\hat{Y}}_{ijk}\;\cdots ,{\hat{Y}}_{ijB_{j}}\right]$$

where $j=1\;,\;2\;,\;3\;,\;\cdots \;,\;p_{i}$; $B_{j}$ represents the number of well-matched patterns, and k represents the kth well-matched pattern.

The approach degree between well-matched patterns and pattern to be recognized is represented by vector $C_{ij}$:

(11) $$C_{ij}=\left[c_{ij1}\;,\;c_{ij2}\;,\;c_{ij3}\;\cdots c_{ijk}\cdots \;c_{ijB_{j}}\right]$$

If the number of patterns is considered, the output of the advanced pattern recognition method can be $Y_{i1}$, $Y_{i2}$,…, $Y_{ij}$,…, $Y_{ip_{i}}$, respectively:

(12) $$Y_{ij}=\left[Y_{ij1}\;,\;Y_{ij2}\;,\;\cdots \;Y_{ijk}\;\cdots ,Y_{ijB_{j}}\right]$$

(13) $$Y_{ijk}=\sum_{l=1}^{k}\alpha_{l}{\hat{Y}}_{ijl}$$

(14) $$\alpha_{l}=c_{ijl}/\sum_{q=1}^{k}c_{ijq}$$

The recognition error with the different number of sensors and the different number of patterns can be represented as:

(15) $$e_{ijk}=\left|\frac{Y_{ijk}-y}{y}\right|$$

(16) $$e_{iu_{i}v_{i}}=\min(e_{ijk})$$

where $u_{i}$ is the determined number of sensors, and $v_{i}$ is the determined number of patterns.

The input of such a best pattern library for the ith subset of measurements is:

(17) $$X_{iu_{i}}^{\prime }=\left[X_{i1}^{\prime },\;X_{i2}^{\prime },\;\cdots \;,\;x_{iu_{i}}^{\prime }\right]$$

The output of such a best pattern library for the ith subset of measurements is:

(18) $$Y_{iu_{i}}=\left[Y_{iu_{i}1},\;Y_{iu_{i}2}\;,\;\cdots \;,\;Y_{iu_{i}v_{i}}\right]$$

The predicted value for the pattern to be recognized is:

(19) $${\hat{Y}}_{i}=\sum_{l=1}^{v_{i}}\alpha_{l}{\hat{Y}}_{iu_{i}l}$$

(20) αl=ciuil∑q=1viciuiq

The predicted values using m subsets of measurements can be given as ${\hat{Y}}_{1}$, ${\hat{Y}}_{2}$,…, ${\hat{Y}}_{i}$,…, ${\hat{Y}}_{m}$, respectively.

### 2.4. Pattern Recognition Based on Gross Sets of Measurements

The gross set of measurements based on the input of each best pattern library for m subsets of measurements can be represented as:

(21) $$X^{\prime }=\left[x_{11}^{\prime }\;,\;x_{12}^{\prime }\;,\;\cdots \;,\;x_{1u_{1}}^{\prime }\;,\;x_{21}^{\prime }\;,\;x_{22}^{\prime }\;,\;\cdots \;,\;x_{2u_{2}}^{\prime },\;\cdots \;,\;x_{m1}^{\prime }\;,\;x_{m2}^{\prime }\;,\;\cdots \;,\;x_{mu_{m}}^{\prime }\right]$$

The predicted value by considering the best number of sensors and patterns is:

(22) $$\hat{Y}=Y_{uv}$$

where u and v are the best number of sensors and patterns when the gross set of measurements is used.

## 3. The Simulation on the Stress Recognition of Shell-Like Structures

### 3.1. The Main Parameters of Shell-Like Structures

**Figure 1:**
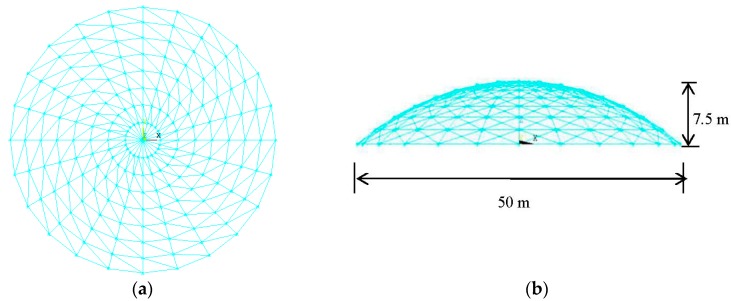
The finite-element model. (**a**) The top view of model; and (**b**) the side view of model.

The finite-element model is built based on the Schwedler shell-like structure [20], which is shown in Figure 1. The shell-like structure is 50 m long, 50 m wide, and 7.5 m high. The uniform mass is 200 Kg/m^2^. The material of the steel members is Q235, the elastic modulus is $2.06\times 10^{11}\;N/m^{2}$, the Poisson ratio is 0.3, and the damping ratio is 0.02 in Rayleigh damping. The section of the member in the radial direction is ϕ133 × 4, while the section of the members in the circle direction and diagonal direction is ϕ127 × 3. The Poisson ratio μ is 0.3. The type of support is a fixed-hinge support.

In this finite-element method calculation, the Karman spectrum is selected to simulate the along-wind fluctuating wind spectrum, while the calculation equation proposed by Panofsky is selected to simulate the vertical-wind fluctuating wind spectrum. The formulas are shown below:

(23) $$S_{u}(z,\omega )=K\nu_{10}^{2}\frac{200}{2\pi }\frac{z}{\bar{\nu_{z}}}\frac{1}{{\left(1+50\frac{\omega z}{2\pi \bar{\nu_{z}}}\right)}^{5/3}}$$

(24) $$S_{w}(z,\omega )=K\nu_{10}^{2}\frac{6}{2\pi }\frac{z}{\bar{\nu_{z}}}\frac{1}{{\left(1+4\frac{\omega z}{2\pi \bar{\nu_{z}}}\right)}^{2}}$$

where S is the fluctuated wind power spectrum, $\bar{\nu_{z}}$ is the average wind speed at the height of z, ω is the fluctuated angular frequency, K is the roughness coefficient on the ground, $K={\left[k/\ln\left(10/z_{0}\right)\right]}^{2}$, $z_{0}$ is the roughness length, and k is the Kaman constant.

### 3.2. Layout of Sensors

A variable coefficient (VC), a kind of parameter used for measuring and reflecting discrete levels of a set of data, is used to determine the placement of sensors, which is represented as:

(25) $$\gamma =\frac{\sigma }{\mu }$$

where γ is the VC of the items of components, σ is the standard deviation of the items of components, and μ is the average value of the items of components.

A time-domain dynamic analysis of this finite-element method is carried out by setting a fluctuating wind load whose average speed reaches 15 m/s. The total loading time is 10 min and 6000 substeps. For each step, the combined stresses of axial stress and bending stress in Y+ at 1/4 length of the members are extracted. According to the values of VC with descending order, the first 26 members are chosen to locate the sensors and extract the stresses; the number of the members are 153, 152, 154, 168, 151, 155, 150, 129, 128, 156, 130, 127, 149, 164, 163, 131, 165, 162, 126, 161, 166, 132, 105, 140, 157, and 139. According to the value of VC with descending order, the first eight points are chosen to extract the displacements, and the number of the members are 2, 12, 13, 14, 15, 16, 17, and 18.

**Figure 2:**
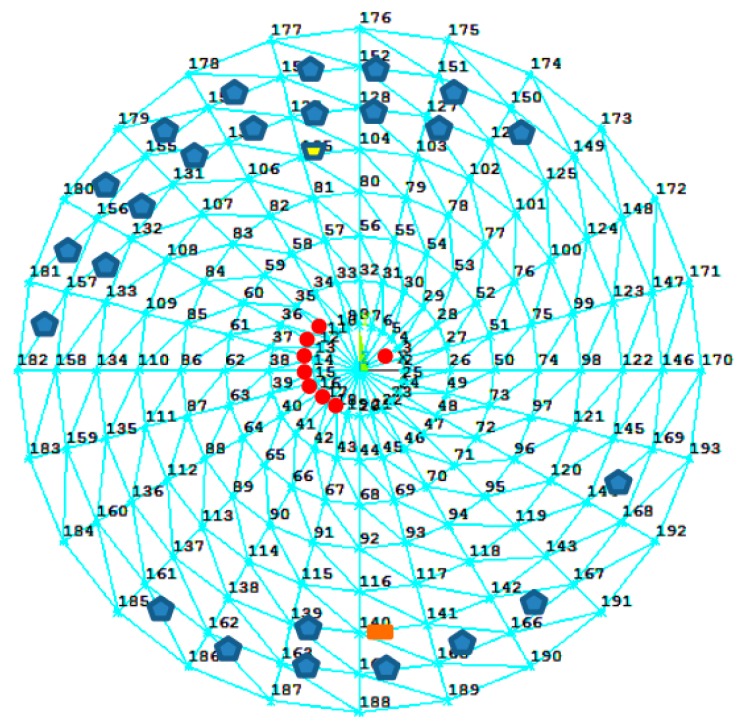
The location of sensors and waited-identification rods. 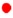 represents the locations of the displacement sensors, 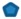 represents the locations of the strain sensors, 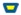 represents Position A, and 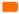 represents Position B.

Considering the values of VC, stress changing, and average stress, the members with No. 105 and No. 140 are selected as the stress of members to be identified, which are denoted as Position A and Position B, respectively. Those positions, the members for extracting stresses, and the points for extracting displacements are shown in Figure 2.

### 3.3. Pattern Sets and Subsets of Measurement

A fluctuated wind load whose average value increases from 5 m/s to 50 m/s (5 m/s for each step) is simulated for the finite-element method analysis. In addition, the stresses and displacements are extracted from the finite-element method every 0.1 s, and the total analysis time is 10 min; that is, the stresses and displacements extracted from the finite-element method analysis are 6000 time steps. Furthermore, the data are separated into the pattern library, testing library, and observed library. The stress and displacement responses from the first time step and those in every three time steps are the data in the pattern library; there are a total of 15,000 patterns. The stress and displacement responses from the second time step and those in every 120 time steps are the data in the testing library; there are a total of 500 patterns. The stress and displacement responses from the third time step and those in every 600 time steps are the data in the observed library, and there are 100 patterns. The subsets of measurement are given in Table 1.

### 3.4. Determination of the Optimal Number of Sensors and Patterns

There are four subsets of measurement, while the determination of the optimal number of sensors and patterns is illustrated using Subset 1.

**Figure 3:**
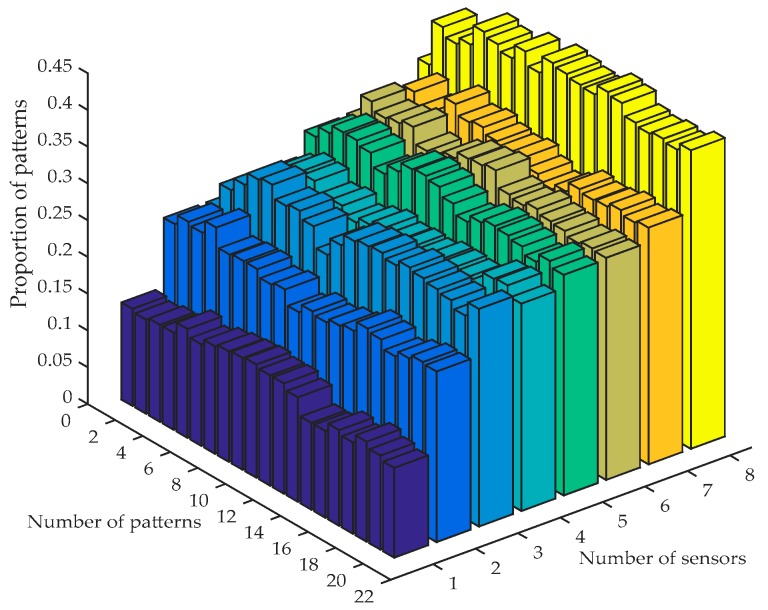
Proportion of patterns with stress error of Position A less than 15%.

(1) The correlation between the responses of the points in No. 1 subset of measurement and the stresses of Position A can be calculated by Equation (4), sorted and shown in Table 2.

(2) The scenarios with different numbers of sensors and patterns are calculated, the pattern with the stress error of Position A less than 15% is signed, and the property of such a pattern is shown in Figure 3.

(3) There are five optimal combinations including the number of sensors and patterns through Figure 3, which are listed in Table 3. By comparing the five combinations in different error ranges, the fourth scenario is chosen as the optimal parameter for identify the stress of Position A. For all the four subsets of measurement, the optimal parameter for identifying the stress of Position A and Position B can be also obtained and are shown in Table 4 and Table 5. According to the determination process of the number of sensors, recognition parameters in two positions with the whole pattern libraries can be determined, and they are listed in Table 6.

## 4. Discussion and Analysis

### 4.1. Comparison of Precision between Distributed and Traditional Concentrated Methods

Through the data of the testing library, stresses of Positions A and B are recognized by the proposed distributed method (DM) and the traditional concentrated method (CM). Table 7 and Table 8 provide various error ranges in different working conditions.

From Table 7 and Table 8, it can be seen that the optimised method is better in the number of working conditions whose errors are less than 5%, 10%, and 15% compared with the traditional CM. The recognition precision of DM is higher than that of CM.

### 4.2. Comparison and Analysis of the Results of Distributed and Concentrated Computing

In this section, the proposed distributed method and the traditional concentrated method are compared by considering the noise interferences.

#### 4.2.1. Noise Analysis

Noise level can be defined as the ratio of root-mean-square of noise to that of the time series:

(26) $$\zeta =\frac{\delta }{\delta^{\prime }}\times 100\%$$

where δ is the root-mean-square of noise, and $\delta^{\prime }$ is the root-mean-square of the time series.

#### 4.2.2. Noise Interferences on All Observed Sensors

**Figure 4:**
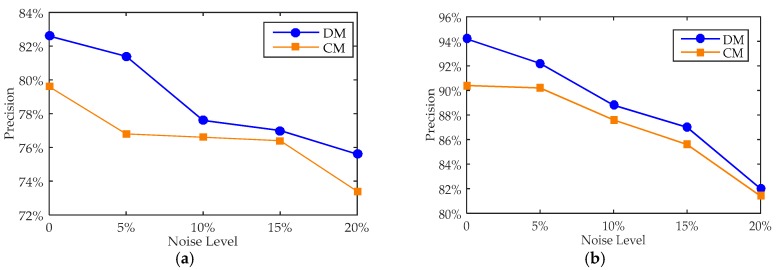
The precision comparison between DM and CM. (**a**) In Position A; and (**b**) in Position B.

When all the observed measurements are affected by different levels of noise interference, identified stress values of Positions A and B using the proposed distributed method and the traditional concentrated method are calculated. The proportions of the members with a stress error less than 15% are shown in Figure 4.

As shown in Figure 4, compared with the proposed distributed method and traditional concentrated method, the distributed method can perform the stress identification with errors less than the traditional concentrated method; in other words, the proposed distributed method cannot only identify the stress, but also realize the distributed calculation to improve the reliability of the system.

#### 4.2.3. Noise Interferences on a Single Sensor

**Figure 5:**
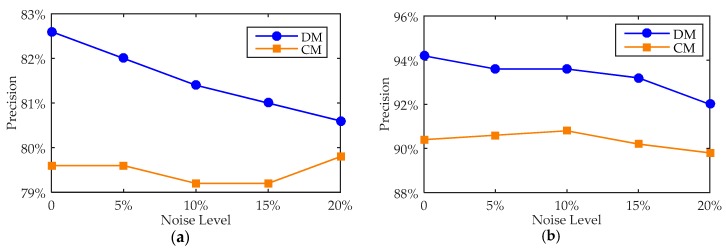
The precision comparison between DM and CM. (**a**) In Position A; and (**b**) in Position B.

There are two scenarios: one is that the sensor located on member No. 152 is subjected to noise, which is used to identify the stress of Position A; the other one is that the sensor located on member No. 161 is subjected to noise, which is used to identify the stress of Position B. The error analysis is shown in Figure 5.

As shown in the Figure 5, compared with the not-optimised DM and CM methods, when one sensor is disturbed by noise at various levels, the recognizing precision of DM is improved clearly on both positions. In summary, regardless of how many sensors are disturbed by noise, the DM has more advantages than CM in stress recognition.

## 5. Stress Recognition Based on Measurements from a Real-World Structure

### 5.1. Introduction of the Structure

**Figure 6:**
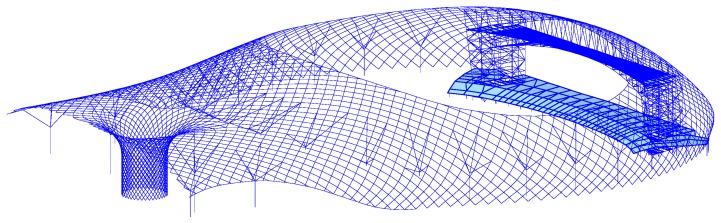
Finite-element method model of the Shenzhen Bay Stadium.

**Figure 7:**
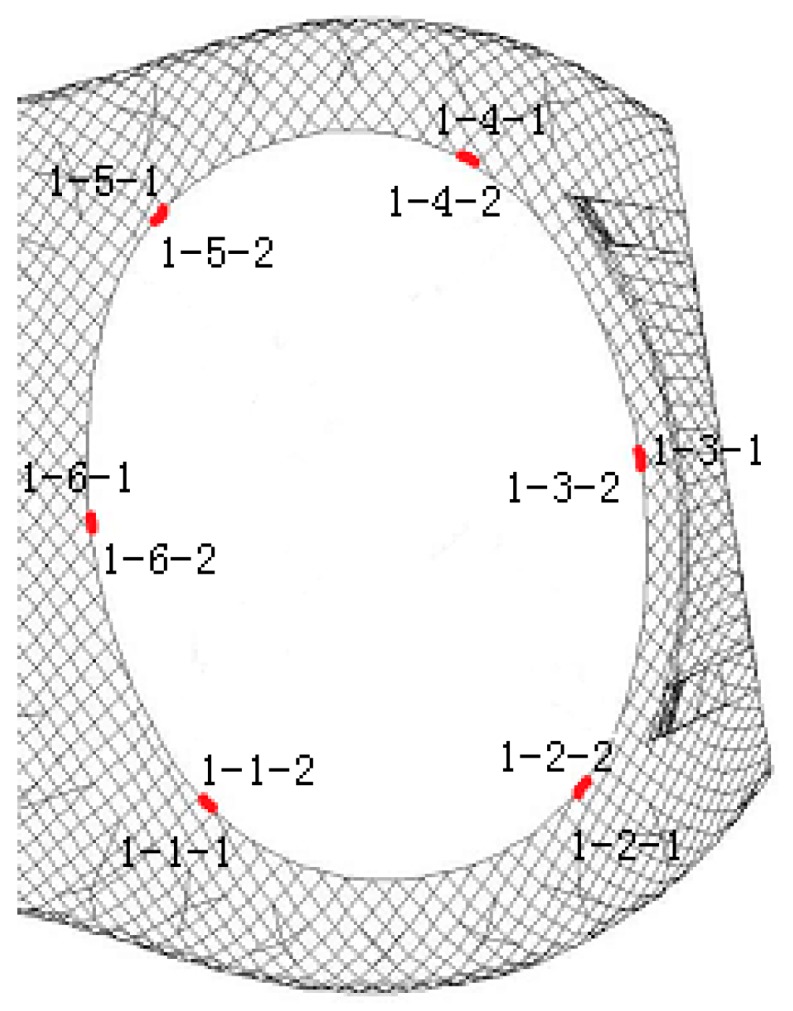
Identification numbers and distribution of sensors.

The Shenzhen Bay Stadium was constructed for the 26th Summer World University Games. It consists of a single shell, with double-layer space trusses, and the vertical support system. The finite element model using Midas^®^ is shown in Figure 6. The strain monitoring arranged at the ring members of the front stadium structure are used for stress recognition. In the construction phase, six members are chosen for monitoring, and each of them has one vibrating string strain sensor placed on both sides. The identification number and distribution of the strain sensors are shown in Figure 7 [11,21].

### 5.2. Pattern Sets

The model can be analysed by instantaneous time-domain analysis in Midas with Earth pulsation, which is simulated by white noise. The amplitudes of accelerations in three orientations X, Y, and Z are 0.1 g, 0.1 g, and 0.15 g, respectively. The frequency range is from 0.5 Hz to 20 Hz and the time range lasts 600 s with a substep of 0.02 s. The stress recognition is that of location 1-2-2 using the other stress measurements.

The stress values extracted from the Midas finite-element method are used to build the pattern library and the testing library, while the stress measurements from the structural health monitoring system of Shenzhen Bay Stadium are used to build the observed library, shown in Table 9.

### 5.3. Comparison of the Results of Two Stress Recognition Methods

The measurements from the structural health monitoring system are less than those for the simulation example of the shell-like structure, so the comparison of the results is between two scenarios: scenario 1 is the method proposed where the optimal number of sensors and fusion patterns is considered, while scenario 2 is the traditional method where the number of sensors and fusion patterns are fixed. The comparison of the recognition results of the two scenarios is shown in Table 10.

From Table 10, it can be found that the recognition error of Scenario 1 is more stable than that of Scenario 2. The proposed method considering the optimal number of sensors and fusion patterns can be effectively used in real-world projects when the measurements include unknown noise interference. The errors are large; this is mainly because the pattern library is based on the simulation data, where there are errors in the finite-element model. However, the performance of stress identification of this real-world application using the proposed distributed method is better than that of the traditional concentrated method.

## 6. Conclusions

Stress prediction for the distributed structural health monitoring using existing measurements and pattern recognition is proposed in this paper. The proposed method considered not only the optimal number of sensors, but also the number of fusion patterns. The traditional pattern recognition method is the concentrated method, while the recognition process is divided into several sub-pattern sets, and the calculation is separated. The stress recognition simulation based on the shell-like structure is given, by which the proposed method was proved to be effective, and the precision was influenced by the different noise levels. In addition, the stress measurements collected from the structural health monitoring of the Shenzhen Bay Stadium are used to verify the stability of the proposed method. 

## Figures and Tables

**Table 1:** The sensors for each subset of measurement.

No. of Subset of Measurements	No. of Members or Points							
1 (displacements)	2	12	13	14	15	16	17	18
2 (stresses)	139	161	162	163	164	165	166	168
3 (stresses)	129	130	131	132	154	155	156	157
4 (stresses)	126	127	128	149	150	151	152	153

**Table 2:** Descending rank in the absolute value of correlation.

Sensor Number	2	12	13	14	15	18	16	17
Absolute value of correlation	0.7657	0.3804	0.3252	0.2892	0.2674	0.2599	0.2560	0.2536

**Table 3:** The number of patterns with the stress identification error less than 15%.

Parameter (Optimal Number of Sensors and Patterns)	Error Range					
	<5%	5–10%	10–15%	15–20%	20–25%	>25%
(8,8)	64	65	76	60	39	196
(8,10)	65	75	68	49	55	188
(8,11)	66	69	70	58	43	194
(8,14)	66	69	75	58	43	189
(8,15)	65	70	73	58	41	193

**Table 4:** Parameters of primary agents in Position A.

Subset of Measurements	Number of Sensors	Number of Fusion Patterns
No. 1 subset of measurements	8	14
No. 2 subset of measurements	8	12
No. 3 subset of measurements	8	13
No. 4 subset of measurements	8	19

**Table 5:** Parameters of primary agents in Position B.

Subset of Measurements	Number of Sensors	Number of Fusion Patterns
No. 1 subset of measurements	8	18
No. 2 subset of measurements	7	18
No. 3 subset of measurements	8	17
No. 4 subset of measurements	7	17

**Table 6:** Recognition parameters in two positions with the set of measurements.

Recognition Positions	Number of Sensors	Number of Fusion Patterns
A	11	19
B	6	12

**Table 7:** Precision comparison for two recognizing ways in Position A.

Recognition Method	Error Range					
	<5%	5–10%	10–15%	15–20%	20–25%	>25%
DM	189	140	84	33	23	31
CM	169	121	97	40	34	39

**Table 8:** Precision comparison for two recognizing ways in Position B.

Recognition Method	Error Range					
	<5%	5–10%	10–15%	15–20%	20–25%	>25%
DM	277	149	45	15	6	8
CM	227	152	65	29	13	14

**Table 9:** Stress measurements in the observed library (MPa).

Sensor Locations	Year 2012							Year 2013	
	March 19	April 23	May 30	July 26	September 7	October 19	December 19	January 23	March 10
1-1-1	42.4	42	40.5	39	39.1	39.5	36.6	38.9	35.2
1-1-2	−32.1	−29.5	−31.5	−30.4	−31.6	−31.9	−34.5	−34.3	−35
1-2-1	−39.1	−38.1	−38.1	−37.9	−38.1	−38.1	−38.1	−38.1	−37.9
1-2-2	41.5	40.7	40.7	40.4	40.7	40.7	40.7	38.9	41.6
1-3-1	−60.5	−67.6	−61.9	−63.9	−63.4	−67.2	−67.7	−67.4	−66.6
1-3-2	−38.9	−38.2	−36.4	−38.2	−38.2	−39.1	−24	−38.2	−38.4
1-4-1	−14.6	−14.4	−14.9	−14.7	−16.1	−16.8	−20	−17.9	−20.1
1-4-2	−44.5	−42.7	−43.3	−44.5	−44.6	−46.8	−48.4	−47.4	−48
1-5-1	−13.9	−14.3	−16.4	−16.3	−18	−17.7	−19.9	−17.9	−22.2
1-5-2	15.9	17	15.1	14.7	14.7	13.2	9.1	15.2	11.5
1-6-1	−81.2	−79.4	−80.5	−81.8	−81.9	−83.7	−85	−82.4	−82.5
1-6-2	−57.3	−55.8	−57.6	−57.1	−59	−60.4	−61.2	−61.3	−63.2

**Table 10:** Comparison of recognition results of two scenarios based on measured data.

Scenario 1 (MPa)	Scenario 2 (MPa)	Measured Data (MPa)	Error of Scenario 1	Error of Scenario 2	Error Difference
54.34	47.37	41.5	30.95%	14.14%	16.81%
53.01	55.21	40.7	27.71%	35.66%	−7.95%
54.18	55.76	40.7	33.13%	37.07%	−3.94%
54.79	55.58	40.4	35.63%	37.57%	−1.94%
54.88	55.79	40.7	34.84%	37.08%	−2.24%
54.89	55.98	40.7	34.87%	37.50%	−2.63%
43.22	19.01	40.7	6.20%	53.27%	−47.07%
42.99	48.21	38.9	10.51%	23.94%	−13.43%
48.31	51.07	41.6	16.14%	22.77%	−6.63%
